# Loss of Rictor in tubular cells exaggerates lipopolysaccharide induced renal inflammation and acute kidney injury via Yap/Taz-NF-κB axis

**DOI:** 10.1038/s41420-020-0274-3

**Published:** 2020-05-29

**Authors:** Yuan Gui, Qing Hou, Qingmiao Lu, Chunsun Dai, Jianzhong Li

**Affiliations:** 1 Center for Kidney Disease, 2nd Affiliated Hospital, Nanjing Medical University, 262 North Zhongshan Road, Nanjing, Jiangsu 210003 China; 2 Division of Nephrology, Department of Internal Medicine, Carver College of Medicine, University of Iowa, Iowa City, IA 52242 USA; 3 Department of Nephrology, The First Affiliated Hospital of Soochow University, Suzhou, Jiangsu 215006 China

**Keywords:** Acute kidney injury, Mechanisms of disease

## Abstract

Our previous study demonstrated that the mammalian target of rapamycin complex 2 (mTORC2) signaling alleviates renal inflammation and protects against cisplatin-induced AKI. However, the underlying mechanisms for mTORC2 in regulating renal inflammation in AKI remain to be determined. In this study, we found that lipopolysaccharide (LPS) could activate mTORC2 signaling in NRK-52E cells, and blockage of mTORC2 signaling led to Yap/Taz degradation, which in turn activated NF-κB signaling and induced inflammatory cytokines secretion. Overexpression of constitutively active Taz (Taz-S89A) could attenuate the inflammation-amplified role of mTORC2 blockage. In mouse models, tubule-specific deletion of Rictor had higher blood urea nitrogen level, severe morphological injury as well as more inflammatory cells accumulation compared with those in their littermate controls. Overall, these results demonstrate that mTORC2 signaling protects against renal inflammation and dictates the outcome of AKI by modulating Yap/Taz degradation.

## Introduction

Acute kidney injury (AKI) is an extremely life-threatening clinical syndrome characterized by a rapid decrease in renal function. Tubulointerstitial inflammatory cells infiltration is the main characteristic of AKI. Accumulated evidence unveils that tubular cells play important roles in AKI-related inflammation. Tubular cells are always the initial site of injury caused by hypoxia or ischemia, which in turn secretes inflammatory mediators and recruits inflammatory cells infiltration, exacerbating renal injury^1–4^. However, the mechanisms by which tubular cells trigger kidney inflammation remain unclear.

The evolutionarily conserved Hippo signaling pathway is recognized as a regulator of cell proliferation, organ size, and tissue regeneration. MST1/2, SAV1, LATS1/2, MOB1A/B, Yes-associated protein (YAP) and its paralogue TAZ (also known as WWTR1) consist of the core components of the Hippo pathway. Among them, YAP and TAZ are the key downstream effectors of the Hippo pathway via MST1 and LATS kinases phosphorylation^5–8^. Recently, several studies have uncovered the roles of Yap/Taz in regulating inflammatory and immune responses^9,10^. Deng et al. reported that phosphorylated Yap is sufficient to attenuate inflammatory responses in osteoarthritis by suppressing NF-κB signaling^10^. However, the mechanisms by which NF-κB signaling triggers Yap/Taz inhibiting inflammatory response are not fully clarified.

The mammalian target of rapamycin (mTOR) plays an essential role in regulating cell growth, proliferation, and survival. The serine/threonine kinase mTOR consist of two distinct complexes: mTOR complex 1 (mTORC1) and complex 2 (mTORC2)^11–13^. Rictor is a critical adapter scaffold protein for mTORC2 that can phosphorylate Akt, protein kinase C (PKC), and serum-and glucocorticoid-induced kinases 1 (SGK1)^14,15^, and Rictor-deficient mice showed a remarkably decreased mTORC2 activity^16^. Our previous studies revealed that endogenous Rictor protects against renal inflammation and cisplatin-induced AKI, moreover, Rictor/mTORC2 stimulates Yap/Taz transcription^17,18^. Thus, we predicted that Rictor/mTORC2 may protect against renal inflammation and acute kidney injury via the Yap/Taz-NF-κB axis.

In this study, we demonstrated that Rictor/mTORC2 signaling is crucial for protecting against renal inflammation and lipopolysaccharide (LPS)-induced AKI through inhibiting Yap/Taz degradation and NF-κB nuclear translocation. Our studies unveiled a novel mechanism for Rictor/mTORC2 signaling in regulating renal inflammation and AKI.

## Results

### LPS activates Rictor/mTORC2 signaling in tubular cells

**Fig. 1:**
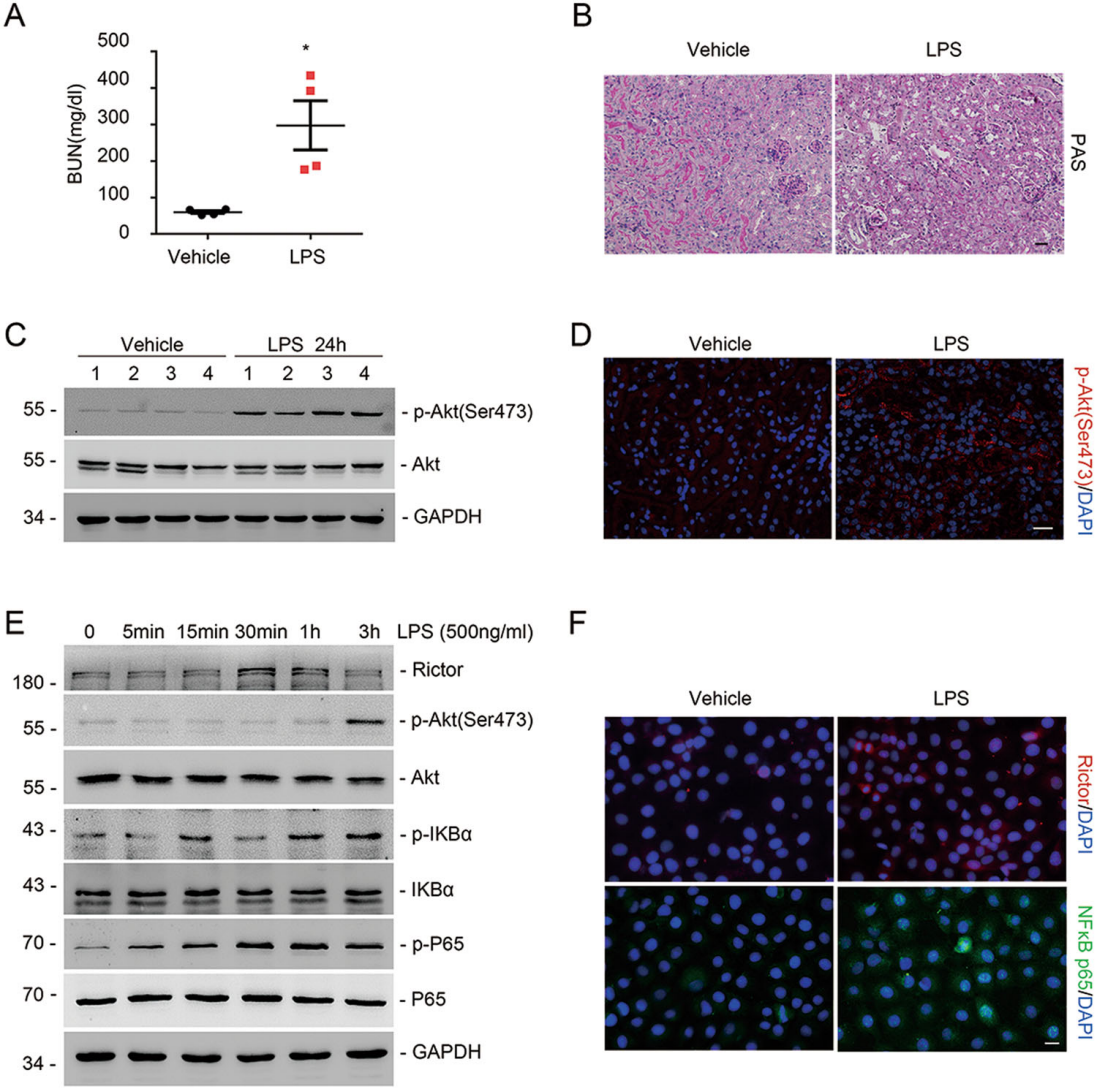
LPS triggers Rictor/mammalian target of rapamycin complex 2 (mTORC2) signaling activation in tubular cells. **a** The graph showing the blood urea nitrogen (BUN) levels in CD1 mice at day 1 after treated with Vehicle or LPS. Each vertical bar represents the mean ± SEM (*n* = 7) analyzed by students *t* test. *P < 0.05 vs. vehicle mice. **b** Kidney histology as shown by periodic acid-Schiff (PAS) staining. Scale bar = 20 μm. **c** Western blotting assay showing the induction of p-Akt (Ser473) in the kidneys with LPS administration compared with those administrated with the vehicle. The numbers indicate the individual animal within each group. **d** Representation images showing the induction of p-Akt(Ser473) in the kidneys at day 1 after LPS. Scale bar = 20 μm. **e** NRK-52E cells were treated with LPS (500 ng/ml) for different time points as indicated. Western blotting analysis showing the induction and activation of Rictor/mTORC2 signaling and NF-κB pathway after LPS treatment in a time-dependent manner. **f** Representation images showing the immunostaining for Rictor and P65NF-κB in NRK-52E cells after LPS treatment. Scale bar = 5 μm.

Tubulointerstitial inflammation is the main characteristic of sepsis-induced AKI^19^. To determine the role of Rictor/mTORC2 signaling in the inflammatory response in AKI, first, we employed a model using LPS injection in CD1 mice. Blood urea nitrogen (BUN) assay and periodic acid–Schiff (PAS) staining revealed LPS-induced mice kidney dysfunction and morphological injury (Fig. 1a–b). Western blot and immunostaining showed that p-Akt (Ser473) was dramatically increased in tubular cells at 1 day after LPS injection (Fig. 1c–d). Then, we stimulated NRK-52E cells, a rat kidney epithelial cell line, with LPS (500 ng/ml) at different time durations as indicated. Western blot assay showed that the abundance of Rictor, p-Akt (Ser473), p-IKBα, and p-P65NFκB were upregulated after LPS treatment (Fig. 1e). Immunostaining for Rictor and P65NF-κB further confirmed these results (Fig. 1f). Taken together, these results suggest that Rictor/mTORC2 signaling is activated in kidney epithelial cells after LPS treatment.

### Specific deletion of Rictor in tubular cells aggravates LPS-induced AKI in mice

**Fig. 2:**
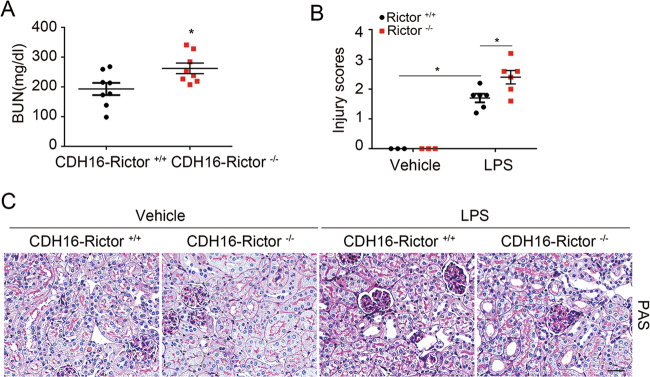
Specific ablation of Rictor in tubular cells aggravates LPS-induced acute kidney injury in mice. **a** The graph showing the blood urea nitrogen (BUN) levels in CDH16^+^-Rictor^+/+^ and CDH16^+^-Rictor^−/−^ mice at day 1 after treated with LPS. Each vertical bar represents the mean ± SEM (*n* = 8) analyzed by students *t* test. *P < 0.05 vs. CDH16^+^-Rictor^+/+^ mice treated with LPS. **c** Kidney histology as shown by PAS staining. Scale bar = 20 μm. **b** The graph showing the injury scores among groups. Each vertical bar represents the mean ± SEM (*n* = 6) analyzed by one-way analysis of variance (ANOVA) followed by post hoc comparisons by Student–Newman–Keuls test. *p < 0.05 compared between different groups.

To elucidate the role of tubular cell mTORC2 activation in LPS-induced AKI, we generated a mouse model with Rictor gene deletion in tubular cells via the Cre-Loxp system as previously reported^18^. Mice were then injected with LPS to induce AKI. As shown in Fig. 2a, the knockouts developed higher BUN levels compared with their littermate controls at 1 day after LPS injection. In addition, loss of brush border, enlargement of tubular lumen, and tubular cell loss were more severe in the KO mice than those in controls (Fig. 2b, c). Thus, these results demonstrated that tubular-specific ablation of Rictor aggravates tubular cell injury and AKI in mice after LPS injection.

### Ablation of Rictor in tubular cells promotes LPS-induced kidney inflammation

**Fig. 3:**
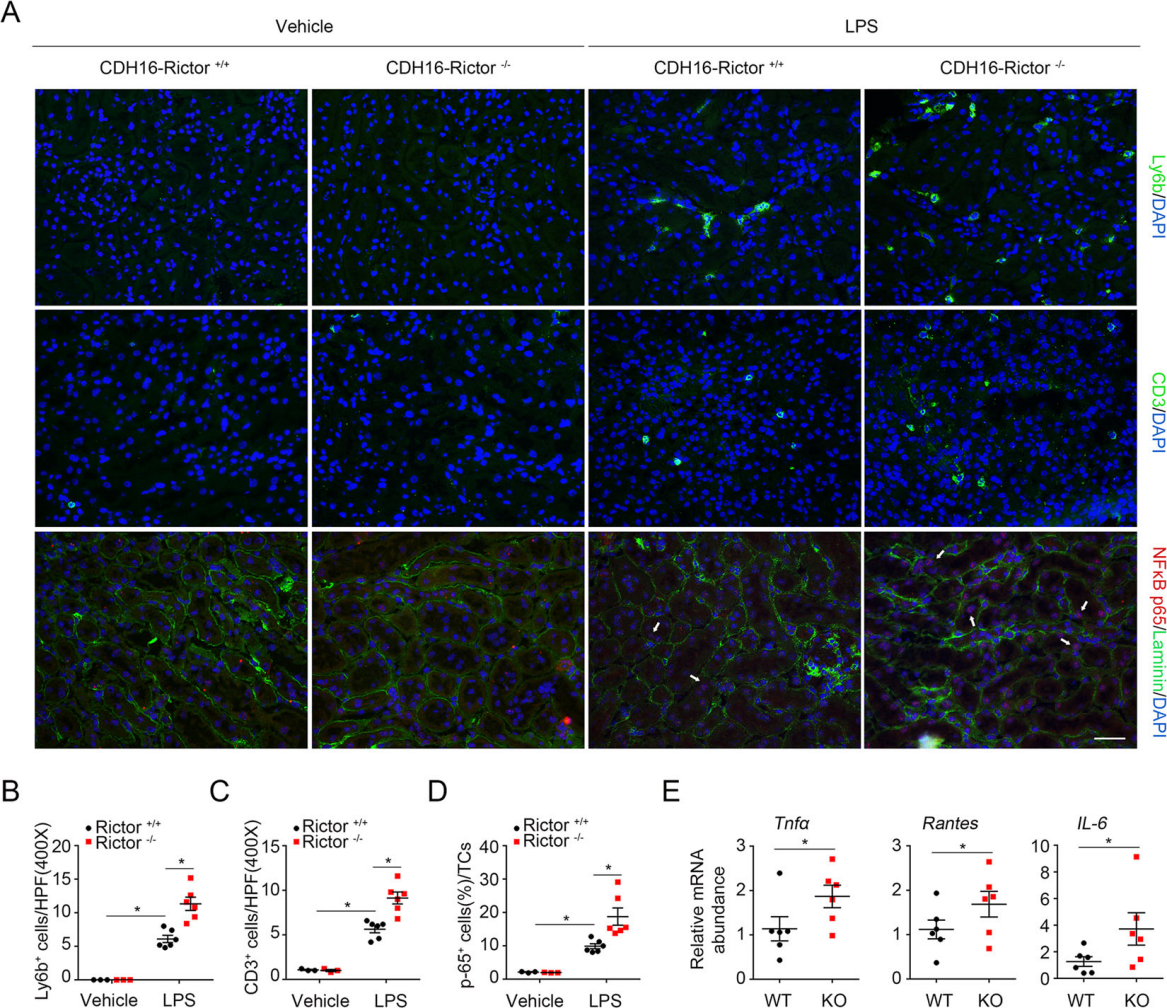
Loss of Rictor in tubular cells aggravates renal inflammation after LPS administration. **a** Representative immunofluorescent staining images for Ly6b, CD3, and P65NF-κB among groups as indicated. White arrows indicate the NF-κB p65-positive tubular cells. Scale bar = 20 μm. **b**–**d** Quantitative determination of Ly6b^+^, CD3^+^, and P65NF-κB^+^ cells among groups as indicated. Each vertical bar represents the mean ± SEM (*n* = 3–6) analyzed by one way analysis of variance (ANOVA) followed by post hoc comparisons by Student–Newman–Keuls test. *p < 0.05 compared between different groups. **e** Real-time qRT-PCR analysis showing the mRNA abundance for *TNF-α*, *Rantes*, and *IL-6* in Rictor^+/+^ and the knockout mice after LPS administration. Each vertical bar represents the mean ± SEM (*n* = 6) analyzed by students *t* test. *P < 0.05 vs. CDH16^+^-Rictor^+/+^ mice treated with LPS.

Considering that inflammation is a crucial component of LPS-induced AKI, we next assessed the role of Rictor ablation in tubular cells on renal infiltration of inflammatory cells after LPS injection. First, we examined the infiltration of renal L6b^+^ neutrophils and CD3^+^ T lymphocytes. At 1 day after LPS injection, the number of L6b^+^ neutrophils and CD3^+^ T lymphocytes significantly increased in the kidney from Tubular-Rictor^−/−^ mice compared with the controls (Fig. 3a–c). In addition, NF-κB nuclear translocation in tubular cells dramatically increased in Tubular-Rictor^−/−^ kidneys compared with the controls (Fig. 3a, d). Second, we examined the expression levels of renal proinflammatory cytokines. Quantitative, real-time RT-PCR (qRT-PCR) showed that the mRNA expression of *TNF-α, Rantes*, and *IL-6* was upregulated in the KO kidneys compared with those from their littermate controls after LPS injection (Fig. 3e). Together, it is concluded that specific deletion of Rictor in tubular cells exacerbates renal inflammation after LPS-induced AKI.

### Blockade of Rictor/Akt axis exacerbates LPS-induced NF-κB signaling activation

**Fig. 4:**
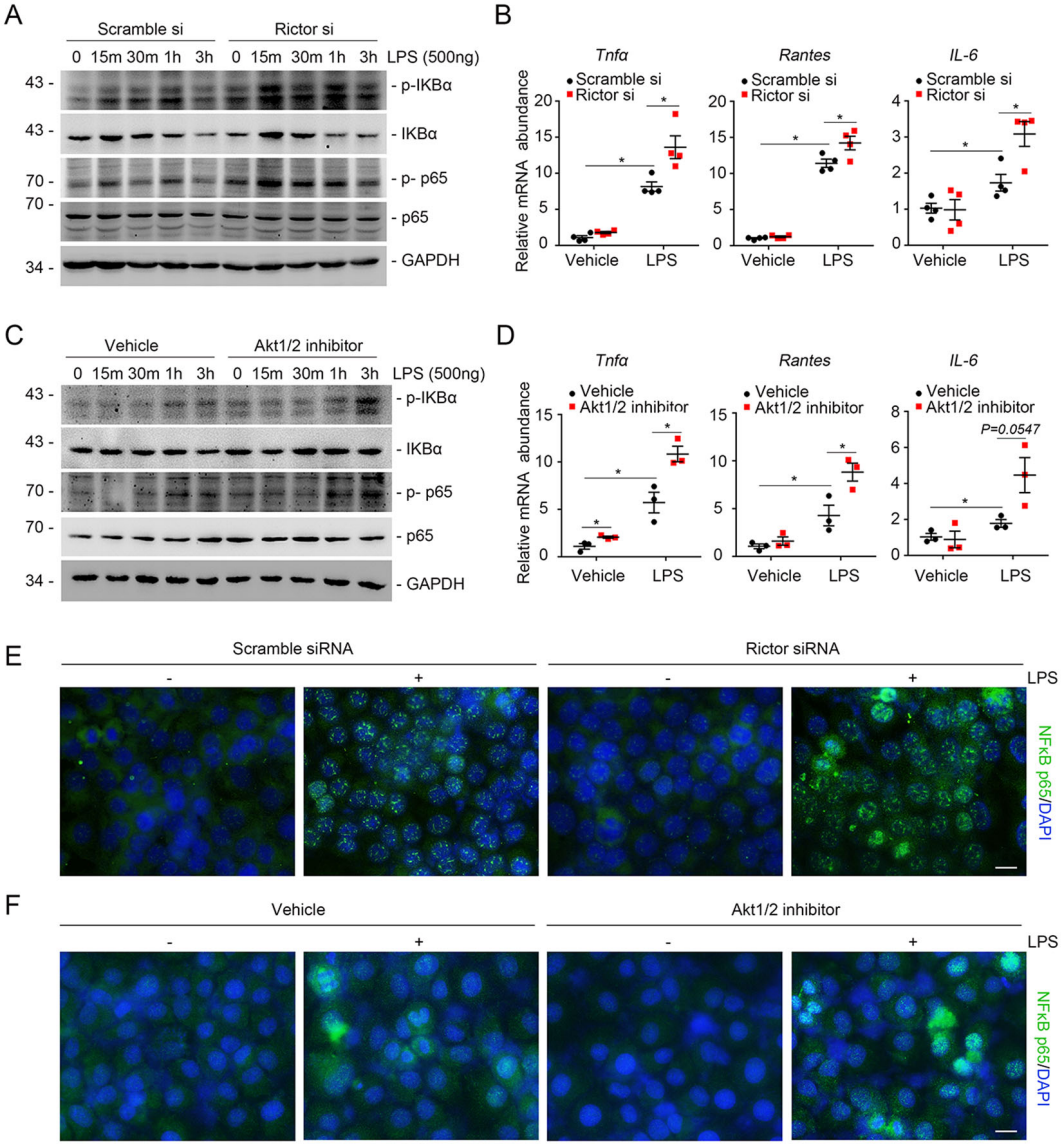
Blockade of Rictor/mTORC2/Akt signaling upregulates LPS-stimulated NF-κB signaling activation. **a** NRK-52E cells were pretreated with scramble, Rictor siRNA for 24 h, followed by LPS (500 ng/ml) treatment for different time points as indicated. Western blotting analysis showing that knocking down Rictor could upregulate LPS-induced NF-κB pathway activation. **b** Real-time qRT-PCR analysis showing the mRNA abundance for *TNF-α*, *Rantes*, and *IL-6* in NRK-52E cells. Each vertical bar represents the mean ± SEM (*n* = 4) analyzed by one way analysis of variance (ANOVA) followed by post hoc comparisons by Student–Newman–Keuls test. *p < 0.05 compared between different groups. **c** Western blotting analysis showing that the abundance for p-IKBα and p-P65NF-κB in NRK-52E cells. **d** Real-time qRT-PCR analysis showing the mRNA abundance for TNF-α, Rantes, and IL-6 in NRK-52E cells. Each vertical bar represents the mean ± SEM (*n* = 3) analyzed by one way analysis of variance (ANOVA) followed by post hoc comparisons by Student–Newman–Keuls test. *p < 0.05 compared between different groups. **e**, **f** Representative micrographs showing immunofluorescent staining for P65NF-κB among different groups as indicated. Cells were co-stained with DAPI (4,6-diamidino-2-phenylindole) to visualize the nuclei. Scale bar = 5 μm.

To elucidate the role of mTORC2 signaling in inflammatory response in vitro, we cultured NRK-52E cells, a rat kidney proximal tubular epithelial cell line, transfected with Rictor small interfering RNAs (siRNAs). Rictor protein expression was downregulated about 70% compared with scramble siRNA (Supplemental Fig. 1A). We then treated the cells with LPS (500 ng/ml) for different durations as indicated. Western blot assay showed that knocking down Rictor expression markedly upregulated LPS-induced NF-κB signaling activation (Fig. 4a). Knocking down Rictor could also upregulate the mRNA abundance of inflammatory cytokines, including *TNF-α, Rantes*, and *IL-6* (Fig. 4b). To decipher the role of Akt, the major downstream molecule for mTORC2 signaling, in regulating NF-κB signaling activation, we also treated NRK-52E cells with Akt1/2 inhibitor to block the Rictor/Akt axis, which was followed by LPS treatment, p-Akt(ser473) expression was markedly suppressed (Supplemental Fig. 1B). Western blot assay and real-time RT-PCR analysis showed that blocking Akt can significantly promote NF-κB signaling activation (Fig. 4c, d). Immunostaining for P65NF-κB further confirmed these results (Fig. 4e, f). Overall, these findings suggest that blockading Rictor/mTORC2 promotes LPS-induced NF-κB signaling activation.

### mTORC2 signaling attenuates the NF-κB pathway by inhibiting Yap/Taz degradation

**Fig. 5:**
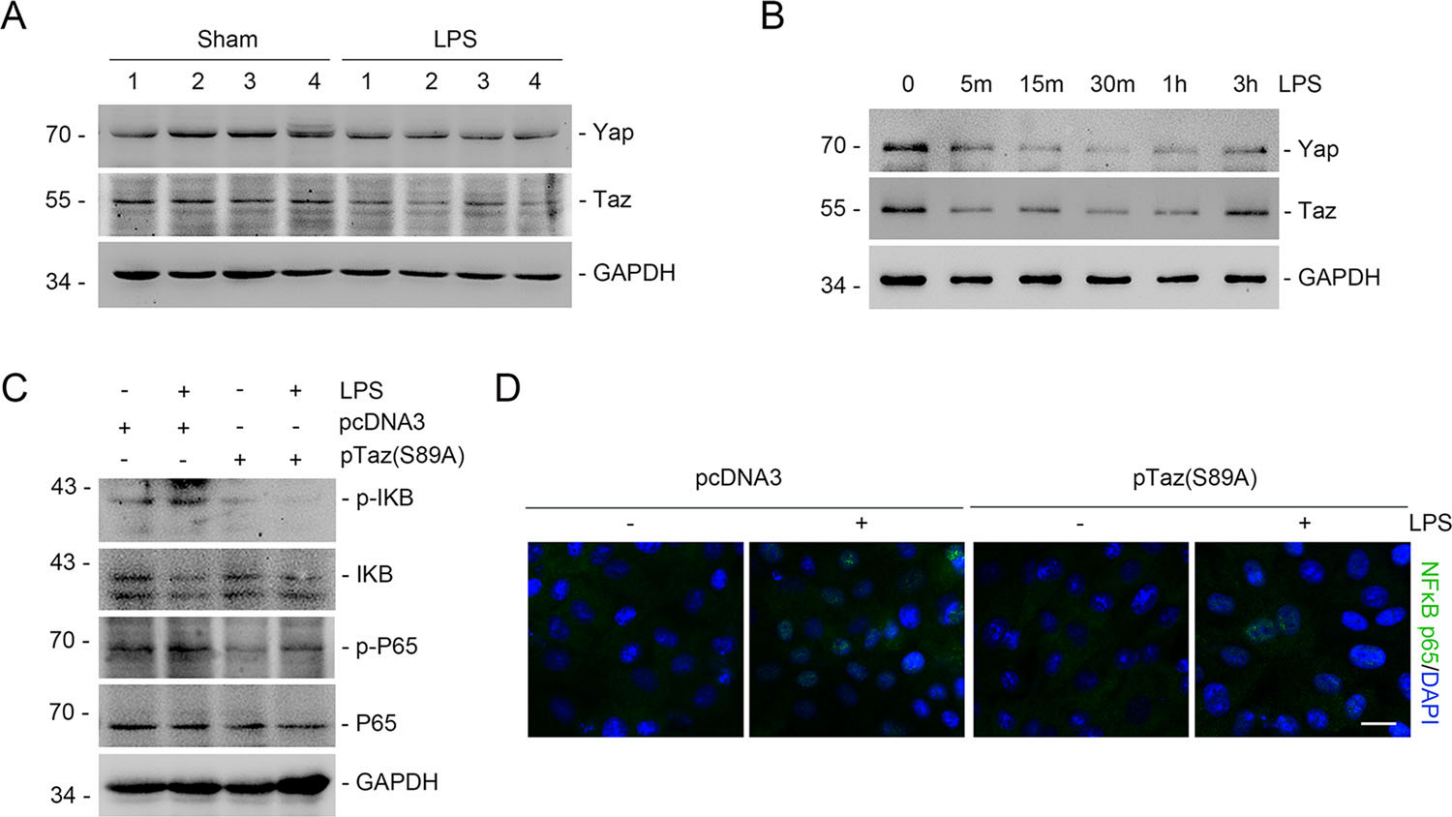
Loss of Yap/Taz mediated NF-κB signaling activation in tubular cells. **a** Western blotting analysis showing that LPS administration could inhibit the expression of Yap and Taz in the kidneys compared with those treated with vehicle. The numbers indicate the individual animal within each group. **b** Western blot analysis of Yap and Taz in NRK-52E cells treated with LPS (500 ng/ml) for the indicated time. **c** NRK-52E cells were transiently transfected with pTaz-S89A for 24 h, followed by LPS (500 ng/ml) treatment. Western blotting assays showing the phosphorylation for IκB and P65NF-κB in NRK-52E cells. **d** Representative micrographs showing the immunostaining for P65NF-κB in NRK-52E cells after various treatments as indicated. Cells were co-stained with DAPI to visualize the nuclei. Scale bar = 5 μm.

Previous studies have demonstrated that Yap/Taz play key roles in regulating inflammatory and immune responses^9^. In addition, Yap can also inhibit TNF-α-induced NF-κB nuclear localization^10^. To verify this finding, we created a model with LPS injection in CD1 mice. Western blot assay showed that the abundance of Yap/Taz was reduced in LPS-administered mice compared with the vehicle control (Fig. 5a). In cultured NRK-52E cells, we also found that Yap/Taz expression was reduced in response to LPS treatment (Fig. 5b). To further decipher the role of Yap/Taz induction in attenuating NF-κB signaling activation, we transfected NRK-52E cells with Taz-S89A expression plasmid. Western blot and real-time RT-PCR results showed that LPS-induced NF-κB signaling activation was considerably compromised in cells expressing Taz-S89A (Fig. 5c), which we further confirmed by immunostaining for P65NF-κB (Fig. 5d). Thus, Yap/Taz acts as an important inflammatory regulator.

**Fig. 6:**
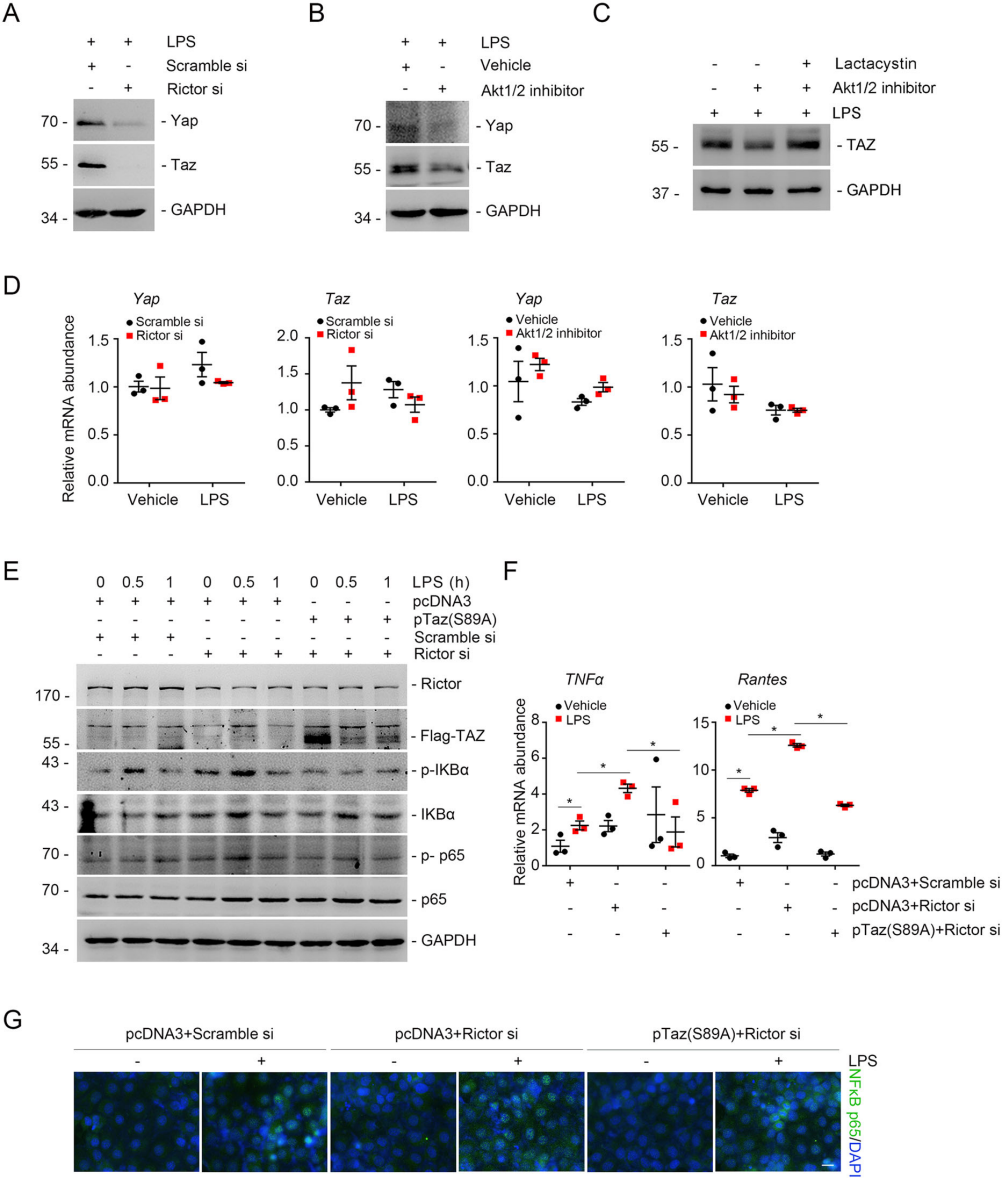
mTORC2 negatively regulates NF-κB pathway by inhibiting Yap/Taz degradation. **a** NRK-52E cells were pretreated with scramble, Rictor siRNA for 24 h, followed by LPS (500 ng/ml) treatment. Western blot analysis showing that knocking down Rictor could reduce Yap/Taz expression. **b** NRK-52E cells were pretreated with Akt1/2 inhibitor for 30 min, followed by LPS (500 ng/ml) treatment. Western blot analysis showed that Akt1/2 inhibitor could suppress Yap/Taz expression. **c** NRK-52E cells were treated with lactacystin, followed by Akt1/2 inhibitor and LPS administration. Western blotting assay showing the abundance for Taz, GAPDH was probed to show the equal loading of the samples. **d** Real-time PCR analysis showing the mRNA abundance for Yap and Taz in NRK-52E cells. **e** Western blotting assays showing the phosphorylation for IκB and P65NF-κB in NRK-52E cells. **f** Real-time qRT-PCR analysis showing the mRNA abundance for *TNF-α*, *Rantes* in NRK-52E cells. Each vertical bar represents the mean ± SEM (*n* = 3) analyzed by one way analysis of variance (ANOVA) followed by post hoc comparisons by Student–Newman–Keuls test. *p < 0.05 compared between different groups. **g** Representative micrographs showing the immunostaining for P65NF-κB in NRK-52E cells after various treatments as indicated. Cells were co-stained with DAPI to visualize the nuclei. Scale bar = 5 μm.

Our published study demonstrated that Rictor/mTORC2 signaling stimulates Yap/Taz transcription in fibroblasts. Therefore, we predicted that in tubular cells, mTORC2 signaling would attenuate the NF-κB pathway through modulating Yap/Taz expression. To verify this supposition, we transfected NRK-52E cells with Rictor siRNA or Akt1/2 inhibitor to block Rictor/mTORC2/Akt axis. We found that blocking the Rictor/mTORC2/Akt axis significantly reduced the total protein levels of YAP and TAZ after LPS treatment (Fig. 6a, b). We also treated NRK-52E cells with lactacystin to inhibit proteasomal degradation. Western blot assay showed that the reduced Taz protein levels were restored when cells were treated with lactacystin (Fig. 6c). In addition, the blockage of the Rictor/mTORC2/Akt axis could not decrease mRNA abundance of Yap/Taz (Fig. 6d). Next, we transfected NRK-52E cells with Taz-S89A to decipher the role of Taz induction in mediating the Rictor/mTORC2-modulated NF-κB pathway. The results showed that expression of TAZ-S89A could largely decrease the LPS-induced overactivation of the NF-κB pathway caused by Rictor siRNA transfection (Fig. 6e, f). Immunostaining for P65NF-κB further confirmed these results (Fig. 6g). Overall, these data demonstrate that mTORC2 signaling attenuates the NF-κB pathway by inhibiting Yap/Taz degradation.

## Discussion

In this study, by employing a mouse model with tubular cell-specific deletion of Rictor, we demonstrated that Yap/Taz is a critical mediator for Rictor/mTORC2 in modulating renal inflammation and LPS-induced AKI. This study deciphers the mechanism for Rictor/mTORC2 signaling in regulating renal inflammation and AKI.

Sepsis-induced AKI manifests as a dramatic decline in the glomerular-filtration rate (GFR), as well as tubular dysfunction. The adaptive responses of tubular cells to an injurious simulation elicit a range of inflammatory mediators from these cells, which results in serious kidney injury^1–4,20–23^. LPS, as an exogenous toxin, is widely used to induce systemic inflammation that mimics many of the initial clinical features of sepsis, which could induce acute kidney dysfunction and inflammatory cells infiltration^24–26^. The NF-κB pathway is a key regulator in the production and secretion of inflammatory cytokines^27–29^. However, the mechanisms by which tubular cells trigger kidney inflammation remain unclear.

It is known that mTORC2 might play a crucial but discrepant role in regulating inflammation and NF-κB signaling in diverse cell types^30–33^. Brown et al. reported that mTORC2 can negatively regulate LPS-induced inflammatory response^34^. Rictor is a defining member of mTORC2 that interacts extensively with mTOR and contributes to mTORC2 assembly^13,35,36^. Our previous study reported that ablation of Rictor in the tubule aggravates inflammatory cells infiltration and kidney injury in cisplatin-induced AKI^18^. In this study, Rictor^fl/fl^ mice were crossed with Ksp1.3/Cre− transgenic mice to generate mouse model with tubular cell-specific deletion of Rictor, in which Cre recombinase is expressed mainly in the collecting ducts, loops of Henle, and distal tubules. In this genetic model, we found ~5% deletion of Rictor in proximal tubules and ~80% deletion in distal tubules^18,37^. LPS-induced nephrotoxicity might involve all segments of the tubule^38^. Therefore, our results suggest a critical role for Rictor/mTORC2 signaling in distal tubules during LPS-induced AKI. As we reported previously, Rictor/mTORC2 is dispensable for tubular cell injury under physiological conditions^18^. Basing from several lines of evidence, we found that Rictor deletion promoted LPS-induced inflammation. The mouse model with tubular cell-specific ablation of Rictor was characterized by more neutrophils and T-lymphocytes accumulation, NF-κB activation in tubular cells, and inflammatory cytokines secretion in the kidneys after LPS injection. In NRK-52E cells, blockage of Rictor/mTORC2 signaling exacerbated LPS-induced inflammatory cytokines secretion. Together, the results suggest that activation of Rictor/mTORC2 signaling is sufficient for preventing LPS-induced renal inflammation and AKI. It should be pointed out that in addition to tubular cells, our published studies reported that Rictor/mTORC2 signaling activation in fibroblasts may protect against tubular cell death and ischemia/reperfusion-induced AKI^39^. However, activation of Rictor/mTORC2 in kidney fibroblasts may lead to interstitial fibrosis in mice^17,40^. Therefore, mTORC2 signaling plays a dual role during kidney injury, and targeting mTORC2 for patients with chronic kidney disease should be cautious.

Hippo signaling is identified to control cell proliferation, organ size, and tissue regeneration^8,41^. Yap and its paralogue Taz are the transcriptional coactivators that are the key effectors of the pathway. Activation of Hippo signaling leads to the phosphorylation of Yap/Taz. By contrast, inactivation of Hippo signaling increases Yap/Taz nuclear localization, where they interact with transcriptional enhancer factor TEF (also known as TEAD) to activate target gene expression, including CTGF, MYC, and Cyr61^5,7^. Substantial evidence demonstrated that NF-κB is recognized as one of the important targets of Yap/Taz, Hippo signaling plays an anti-inflammatory role in regulating innate immunity and autoimmunity^10,42,43^. Moreover, our published study demonstrated that Rictor/mTORC2 signaling stimulates Yap/Taz transcription in fibroblasts^17^. In this study, we found that LPS can decrease the protein expression of Yap/Taz through mTORC2 signaling-mediated Yap/Taz degradation. The conclusion is supported by the following evidence. First, in NRK-52E cells, the expression of Yap and Taz was significantly suppressed by Rictor siRNA or Akt1/2 inhibitor after LPS stimulation. Second, inhibiting proteasomal degradation with lactacystin could abolish Akt1/2 inhibitor-induced Taz degradation after LPS administration. Moreover, blockage of the Rictor/mTORC2/Akt axis could not decrease the mRNA abundance of Yap/Taz. Third, overexpression of constitutively active Taz could largely decrease the LPS-induced overactivation of the NF-κB pathway and inflammatory cytokines secretion caused by Rictor siRNA transfection. However, the underlying mechanisms of Rictor/mTORC2 signaling in regulating the Yap/Taz degradation need further investigation. It should be noted that Rictor/mTORC2 respectively plays transcriptional- or degradational-regulatory roles in fibroblasts and tubular cells, suggesting the distinct regulatory mechanisms in different cell types of the kidney.

In summary, our study demonstrated that Rictor/mTORC2 signaling protects against renal inflammation and LPS-induced AKI through inhibiting Yap/Taz degradation and NF-κB nuclear translocation. Activation of mTORC2 signaling may potentially provide a new strategy for AKI.

## Materials and methods

### Mice and animal models

Mouse model with tubular cell-specific deletion of Rictor gene was generated as previously reported^18^. Briefly, homozygous Rictor floxed mice (C57BL/6J background) from Dr Magnuson (University of Vanderbilt) were bred with the Ksp1.3/Cre transgenic mice from Jackson lab (012237, C57BL/6J background). After several times of cross breeding, mice with genotyping Rictor^fl/fl^, Cre^+/−^ were considered as knockouts. The Age and gender matched Rictor^fl/fl^, Cre^−/−^ mice of the same litter were considered to be the control littermates. Tubule-Rictor−/− or Tubule-Rictor+/+ mice at the age of 6W–8W were selected to establish the AKI model by single intraperitoneally injection of LPS (7.5 mg/Kg). For some experiments, CD1 male mice weighing ~20–25 g were also injected intraperitoneally with 7.5 mg/kg LPS to induce AKI. Mice were euthanized at 24 h after LPS administration. Blood and kidney samples were harvested for further analysis. All of the mice were housed under specific pathogen-free (SPF) facilities in Animal Center of Nanjing Medical University according to institutional animal care guidelines with approval from the Nanjing Medical University’ Institutional Animal Care and Use Committee.

### Cell culture and treatment

NRK-52E cells were maintained in Dulbecco’s modified Eagle’s medium-F12 medium (DMEM-F12) plus 5% fetal bovine serum (Invitrogen, Grand Island, NY) at 37 °C and 5% carbon dioxide atmosphere. NRK-52E cells were purchased from the ATCC (CRL-1571TM, Manassas, VA). Cells were plated onto 6-well plates to 60–70% confluence, the complete medium was changed with medium without serum. LPS was added to the serum-free medium at a concentration of 500 ng/ml for various duration. Akt1/2 kinase inhibitor (cat: A6730, Sigma-Aldrich, St. Louis, MO) was added at 30 min before LPS stimulation, and lactacystin (Santa Cruz Biotechnology, lnc.) was added at 30 min before Akt1/2 kinase inhibitor. NRK-52E cells were treated with Rictor siRNA (Integrated Biotech Solutions, Shanghai, China) and 3XFlag pCMV5-TOPO-TAZ (S89A) kindly provided by Jeff Wrana (Addgene plasmid #24815) using Lipofectamine 3000 reagent (Invitrogen, Grand Island, NY) in accordance with the kit’s protocol.

### Serum BUN assay

Blood urea nitrogen (BUN) was measured using a QuantiChrom Urea Assay kit (cat: DIUR-500, Hayward, CA) as per the manufacturer’s instructions.

### Histology and immunohistochemistry

Mouse kidney specimens were pre-fixed with 10% neutral formalin overnight and were paraffin-embedded. Renal sections (3-µm thick) were prepared for periodic acid–Schiff (PAS) staining. The severity of renal injury was evaluated as follows, tubular necrosis, loss of brush borders, cast formation, and tubular dilatation. A semi-quantitative score was implemented to evaluate each tissue. Accordingly, the injury score was scored with: 0, ≤10% of the injury area stained; 1, 11–25% stained; 2, 26–50% stained; 3, 51–75% stained; and 4, >75% stained. More than ten microscope fields (magnification, ×400) were randomly selected from each section under a light microscope and we calculated the mean score. Slides were captured on a Nikon Eclipse 80i microscope connected to a digital camera (DS-Ri1, Nikon).

### Immunofluorescence

Frozen sections from the kidneys (3 μm) were fixed in 4% paraformaldehyde in PBS for 15 min and washed two times, and then permeabilized for 5 min at room temperature by incubation in 0.5% Triton X-100 in PBS. Blocking was performed in 5% donkey serum in PBS for 50 min at 37 °C, slides were processed for immunostaining against p-Akt (Ser473) (cat: 4060, Cell Signaling Technology), Rictor(cat: ab70374, Abcam), Ly6b (cat: MCA771G, AbD Serotec, Raleigh, NC), CD3 (cat: 555273, BD Pharmingen), NF-κB (cat: 8242, Cell Signaling Technology), Taz (cat: 83669, Cell Signaling Technology). Cells attached on coverslips were washed two times using cold PBS and fixed in a mixture of ice-cold acetone and methanol (1:1) for 10 min at −20 °C and dried in air. Following three washes with 1×PBS, the cells were permeabilized with 1% Triton X-100 for 5 min and blocked in blocking buffer (4% BSA in PBS) for 50 min at room temperature, followed by incubation with anti-NF-κB (cat: 8242, Cell Signaling Technology) and anti-Rictor (cat: ab70374, Abcam), followed by 30 min with TRITC- or FITC-conjugated secondary antibodies. Cell nuclei were also counterstained with 4ʹ,6-diamidino-2-phenylindole (DAPI). Immunofluorescence signals were captured on a fluorescence microscope (Nikon 80i) attached a digital camera.

### Western blot analysis

Protein samples of NRK-52E cells were prepared for SDS-PAGE in 1× SDS sample buffer. The tissue protein was extracted from the kidney and homogenized in RIPA lysis buffer (0.1% sodium dodecyl sulfate (SDS), 1% NP40, 100 μg/ml phenylmethylsulfonyl fluoride) containing a cocktail of protease inhibitor (Sigma, St. Louis, MO, USA) on ice, then the lysates mixture were centrifuged at 13,000 × *g* for 30 min at 4 °C, and the supernatant was collected and stored at −80 °C. A bicinchoninic acid assay (BCA) kit (Thermo Scientific) was applied to quantify protein concentration. Each lane was loaded with equal quantity of protein, subjected to 10 or 15% SDS-PAGE gels, transferred to PVDF membrane and then blocked. The primary antibodies were listed below: anti-Rictor (cat: ab70374, Abcam), anti-phospho-Akt (Ser473) (cat: 4060, Cell Signaling Technology), anti-Akt (cat: 4691, Cell Signaling Technology), anti-GAPDH (cat: FL-335, Santa Cruz Biotechnology, Dallas, TX), anti-Yap (cat: 4912, Cell Signaling Technology), anti-p-NF-κB (cat: 3033, Cell Signaling Technology, USA), anti-NF-κB (cat: 8242, Cell Signaling Technology), anti-p-IκBα (cat: 2859, Cell Signaling Technology), anti-IκBα (cat: 4812, Cell Signaling Technology), and anti-Taz (cat: 83669, Cell Signaling Technology). The signal intensity of protein bands were scanned and quantified by Image J software (NIH).

### RNA extraction and detection of mRNA

Total RNA of cultured cells and mouse kidneys were extracted using Trizol reagent (Invitrogen) according to the manufacturer’s instruction, and concentration was determined by measuring optical absorbance at 260 nm. Subsequently, 1 μg of total RNA was used to synthesize cDNA with a ReverTra Ace qPCR RT Kit (Vazyme, Nanjing, China). Quantitative real-time PCR (qRT-PCR) was carried out to measure gene expression by using an Applied Biosystems 7300 Real-time PCR System and real-time PCR assay (Vazyme). Realtive fold changes were calculated using the 2δCt method, where △CT = CT_gene_ − CT_control_.

### Statistical analysis

The data from the present study are presented as mean±standard error (S.E.M.). SigmaStat software (Jandel Scientific Software) was carried out to perform statistical analysis of the data. The students *t* test was used for comparisons between two groups. One-way analysis of variance (ANOVA) followed by the Student–Newman–Keuls test was assessed for comparisons among multigroup. We regarded a *p* < 0.05 (two-side) as statistically significant.

## Supplementary information

Supplemental Figure1

Supplemental Figure Legend
