# Supplementary figures and images for: Loss of Rictor in tubular cells exaggerates lipopolysaccharide induced renal inflammation and acute kidney injury via Yap/Taz-NF-κB axis
Source: Cell Death Discov. 2020 May 29;6:40. doi: 10.1038/s41420-020-0274-3 (PMC7260239; doi:10.1038/s41420-020-0274-3)

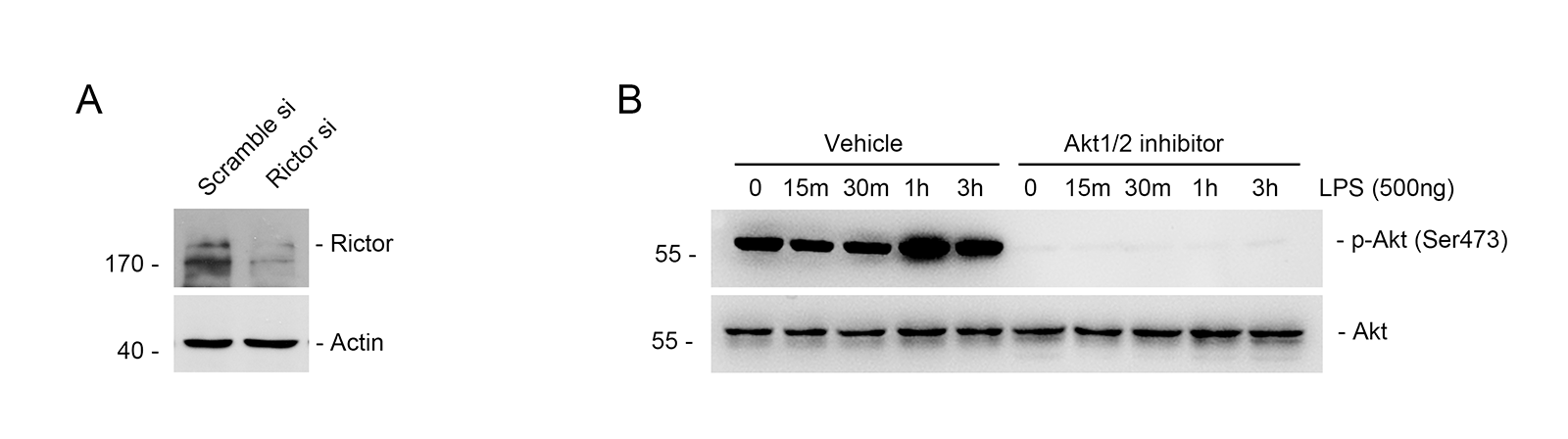

Supplement: Supplementary file 1 — Supplemental Figure1 [file 41420_2020_274_MOESM1_ESM.tif]
