# Supplementary material for: Loss of Rictor in tubular cells exaggerates lipopolysaccharide induced renal inflammation and acute kidney injury via Yap/Taz-NF-κB axis
Source: Cell Death Discov. 2020 May 29;6:40. doi: 10.1038/s41420-020-0274-3 (PMC7260239; doi:10.1038/s41420-020-0274-3)
Supplement: Supplementary file 2 — Supplemental Figure Legend [file 41420_2020_274_MOESM2_ESM.docx]

**Supplemental Figure Legend**

**Supplemental Figure 1. The downregulation of Rictor and p-Akt (Ser473) in NRK-52 cells.** (A) NRK-52E cells were pretreated with scramble or Rictor siRNA for 24 h. Western blotting analyses revealing the downregulation of Rictor protein in NRK-52E cells at 24 h after Rictor siRNA transfection. (B) Western blotting analyses demonstrating the downregulation of p-Akt (Ser473) after Akt1/2 inhibitor treatment.
